# About Making Lignin Great Again—Some Lessons From the Past

**DOI:** 10.3389/fchem.2019.00565

**Published:** 2019-08-29

**Authors:** Wolfgang G. Glasser

**Affiliations:** Sustainable Biomaterials, Virginia Tech, Blacksburg, VA, United States

**Keywords:** biobased polymer, compostable plastics, lignin modification, lignin properties, sustainable thermoplastics, natural adhesives, lignin compatibility

## Abstract

Lignin, the second most abundant biopolymer on the planet, serves land-plants as bonding agent in juvenile cell tissues and as stiffening (modulus-building) agent in mature cell walls. The chemical structure analysis of cell wall lignins from two partially delignified wood species representing between 6 and 65% of total wood lignin has revealed that cell wall-bound lignins are virtually invariable in terms of inter-unit linkages, and resemble the native state. Variability is recognized as the result of isolation procedure. In native state, lignin has a low glass-to-rubber transition temperature and is part of a block copolymer with non-crystalline polysaccharides. This molecular architecture determines all of lignin's properties, foremost of all its failure to undergo interfacial failure by separation from (semi-) crystalline cellulose under a wide range of environmental conditions. This seemingly unexpected compatibility (on the nano-level) between a carbohydrate component and the highly aromatic lignin represents a lesson by nature that human technology is only now beginning to mimic. Since the isolation of lignin from lignocellulosic biomass (i.e., by pulping or biorefining) necessitates significant molecular alteration of lignin, isolated lignins are highly variable in structure and reflect the isolation method. While numerous procedures exist for converting isolated (carbon-rich) lignins into well-defined commodity chemicals by various liquefaction techniques (such as pyrolysis, hydrogenolysis, etc.), the use of lignin in man-made thermosetting and thermoplastic structural materials appears to offer greatest value. The well-recognized variabilities of isolated lignins can in large part be remedied by targeted chemical modification, and by adopting nature's principles of functionalization leading to inter-molecular compatibility. Lignins isolated from large-scale industrial delignification processes operating under invariable isolation conditions produce polymers of virtually invariable character. This makes lignin from pulp mills a potentially valuable biopolymeric resource. The restoration of molecular character resembling that in native plants is illustrated in this review via the demonstrated (and in part commercially-implemented) use of pulp lignins in bio-degradable (or compostable) polymeric materials.

## Introduction

Lignin is well-known as the second most abundant biopolymer on Earth (Freudenberg and Neish, 1968; Sarkanen and Ludwig, 1971; Lewis and Sarkanen, 1998). Except for plants grown under water (or in juvenile condition, such as in annual crops), the planet's method of harnessing solar energy and recycling carbon dioxide, water, and oxygen through photosynthesis involves the formation of lignin. Lignin is always the minor component (by mass) of lignocellulosic plant species, and it plays a major and important role in the mechanical (bonding and stiffening), nutritional, and soil preservation role of nature. The process of lignification, and the structural details of lignin, have been the subject of numerous recent articles, and reviews (Forss and Fremer, 2003; The Ljungberg Textbook, 2008). This paper reviews the issue of lignin's variability, and specific aspects of its utilization potential in structural materials. While lignin has captured major markets as water-soluble derivative for many decades, mostly from sulfite pulping (Gargulak and Lebo, 2000), its potential use from other delignification processes has rarely extended beyond that as process-fuel. However, the quest for using renewable resources in place of fossil carbon sources for chemicals and materials has recently led to an accelerated exploration of lignin (and other biomass sources) in structural polymers (Doherty et al., 2011; Thakur et al., 2014; Upton and Kasko, 2016; Graichen et al., 2017). The many options investigated for lignin include chemical products derived from numerous depolymerization techniques (Zakzeski et al., 2010; Xu et al., 2014; Beckham et al., 2016; Kozliak et al., 2016; Cheng et al., 2018; Sun et al., 2018; Van den Bosch et al., 2018); from polymer fractionation using solvents and/or membrane filtration (Huang et al., 2017; Li and Takkellapati, 2018); from thermal conversion (carbon fibers; 3-D products; etc.) (Baker and Rials, 2013; Li et al., 2017); from chemical modification (Wang et al., 2016; Mueller et al., 2019); and many others. Thermosetting and thermoplastic materials have attracted special interest due to their market value and size, but they also present the most formidable obstacles since most natural polymers are neither soluble nor thermally deformable.

This review will attempt to illustrate the fundamental principles of how nature's approach to the assembly of recyclable but interfacial-failure-proof materials can (a) be adopted for the design of structural materials involving lignin; and (b) how these materials can draw specific benefits from lignin's original structural design and properties. Many of these principles have been the result decades ago of studies focused on both biochemical (i.e., wood formation and thus lignification) and papermaking (and thus delignification) techniques, which constitute the basic roots of our understanding of lignin without being readily connected to present-day polymer and materials science literature.

## Structural Aspects

**Figure 1:**
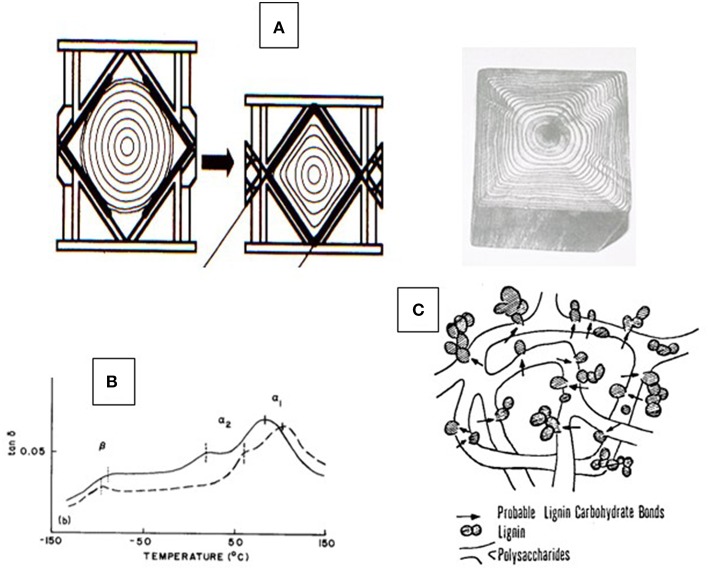
Aspects of thermal properties of wood—**(A)** Illustration of the irreversible thermal deformation of a moisture-plasticized tree trunk under the influence of high-pressure steam and pressure (Ito et al., 1998); **(B)** Glass-to-rubber transitions (T_g_) of two moisture-plasticized solid wood slices (spruce and beech) recorded by dynamic mechanical thermal analysis (DMTA) revealing the T_g_ of native lignin (α_1_), native carbohydrates (α_2_), and moisture exchange (β) (Kelley et al., 1987); and **(C)** generic proposed model of the lignin-carbohydrate copolymer architecture according to Košikova et al. (1978).

Lignin is formed by the *in-situ* polymerization of a mixture of *para*-hydroxy cinnamyl alcohols with an enzyme-triggered mechanism involving free radicals. In native plants, lignin's aromatic structure is dominated by alky-aryl ether bonds that accommodate a limited variability in relation to precursor supply, which represents a mixture of three *p*-hydroxy cinnamyl alcohols (also called C_9_-species), designated as coumaryl (*p-*OH), coniferyl (Gua), and sinapyl (Syr) alcohol (Freudenberg and Neish, 1968). The enzymatic electron-abstracting (oxidation) reaction of the phenolate species generates a series of free phenoxy radical species that exist in a variety of mesomeric forms. The phenoxy radicals react (“couple”) with one another either in juvenile plant tissue, which is in need of immobilizing (immature) cells with respect to each other (i.e., bonding), or in mature tissue, which is in need of cell wall stiffening. This process has been studied (experimentally and by computer simulation) and described abundantly (Glasser and Glasser, 1974, 1981; Glasser, 1981; Glasser et al., 1981; Lewis and Sarkanen, 1998). It results in a chemical structure with many different inter-monomer bonds (also called “inter-unit bonds”) that are the consequence of the presumed randomness of the bond formation between the different mesomeric forms of the phenoxy radicals. It has recently been established that lignin in different tissue locations (such as stalk vs. endocarp, middle lamella vs. cell wall, etc.) can vary significantly, esp. in relation to Syr:Gua ratio (Rencoret et al., 2018). This creates the possibility of different functionalities in different tissue locations. However, it is well-established that all lignins, regardless of their monomer composition (i.e., “precursor ratio,” *p*-OH: Gua: Syr), have a preponderance of alky-aryl ether inter-monomer bonds that amounts to around 50–60% of bond-total in native wood, and it reaches as high as 84% in some other plants (Rencoret et al., 2018). The ether content rises with the methoxy content on the aromatic ring of the monomer species (Glasser et al., 1983b). Because of this linkage distribution, the following corroborating observations can be made:

1. An analytical procedure most qualified to depolymerize lignin by severing exclusively alkyl-aryl ether linkages (i.e., no side-reactions), produces a mixture of building blocks that consists predominantly of monomers (50%), dimers, and some oligomers (Nimz and Das, 1971; Lapierre et al., 1986).
2. Since lignin's average degree of dehydrogenation (i.e., the number of sites per C_9_-unit being linked by the coupling of free radicals produced by dehydrogenation) is <2.00/C_9_ (Freudenberg and Neish, 1968; Glasser and Glasser, 1976), lignin can be considered a predominantly linear chain of monomeric, dimeric, and oligomeric phenylpropane units having molecular sizes of <1,000 g/M that are linked in alkyl-aryl ether fashion (Funaoka, 2013).
3. The concentration of phenolic OH-groups in native lignin is exceedingly low (<0.25/C_9_).
4. Lignin has a low glass-to-rubber transition point, T_g_, making wood thermally-deformable as long as plasticization by water is maintained at temperatures exceeding 100°C as shown in Figures 1A,B, i.e., under pressure (Kelley et al., 1987; Ito et al., 1998).
5. There is significant variability in bond types, in addition to different alky-aryl ether types, between monomeric C_9_-species that have the potential of influencing thermal, and solubility properties.

**Figure 2:**
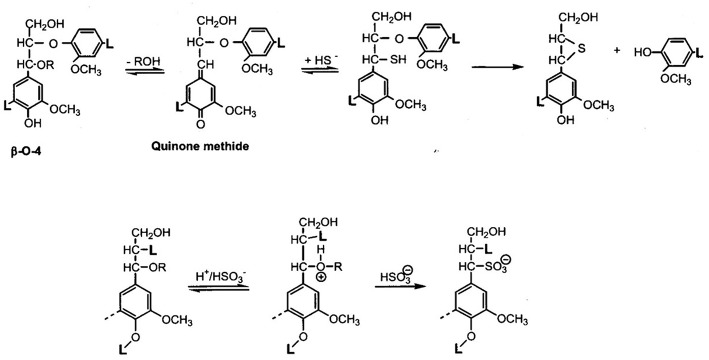
Main reaction mechanisms of lignin during pulping—TOP: Alkyl-aryl ether cleavage and phenolic OH-generation via quinonemethide intermediates in alkali; the involvement of bisulfide ions prevents dissolution-defeating side-reactions from taking place, leading to increased alkali-solubility and effective depolymerization; BOTTOM: Formation of (water-soluble) lignin sulfonates via benzylium ion intermediate aromatic structures in pulping reactions under acidic conditions with bisulfite.

This understanding has given rise to the picture of the amorphous component of wood being a block copolymer of modestly sized lignin phases with non-crystalline hetero-polysaccharides (Figure 1C) (Košikova et al., 1978; Koshijima and Watanabe, 2003). That the inherently thermoplastic lignin component of the native block copolymer will undergo phase separation and relocation in the cell wall via melt-state after exposure to high temperature (224°C) treatment for short periods of time (180 s) without rapid steam decompression (“explosion”) was demonstrated by Debzi et al. (1991) and others. When subjected to delignification treatments during pulp production, wood is exposed to aqueous acid or alkali (Rydholm, 1965; The Ljungberg Textbook, 2008). This fractionation proceeds via two primary reactive lignin intermediates, benzylic cations (in acid), and quinonemethides (in alkali) (Figure 2). In acid, fractionation is made possible by the introduction of solubilizing (in water) functional (sulfonate) groups in the lignin polymer without (significant) depolymerization (except for the most labile benzyl-ether bonds) while severing lignin-carbohydrate bonds by hydrolysis; and in alkali, lignin removal requires depolymerization along the alky-aryl ether chain with simultaneous generation of (acidic) phenol groups (Sarkanen and Ludwig, 1971; The Ljungberg Textbook, 2008). This depolymerization is assisted catalytically by the presence of sulfide and hydrosulfide ions in the pulping liquor, which become the preferred reaction partner for the quinonemethide intermediates (Figure 2). Both delignification options encounter competitive secondary (condensation) reactions by internal competition for the reactive intermediates, benzylic cations and quinonemethides, thereby generating new carbon-to-carbon bonds. This is particularly significant in acid sulfite pulping where, for example, the indigenous presence of pinosylvin -an aromatic extractive substance with resorcinol structure in the heartwood tissue of pines- out-competes sulfite ions for the reaction with cations, leading to water insolubility of the modified (condensed) lignin (Hillis, 1962). This illustrates the virtual inescapability of lignin structure modification during aqueous pulping resulting in newly created structural variability. That this variability arises *after* lignin removal from cell tissue, in aqueous solution, becomes evident with the analysis of residual lignin isolated from incompletely delignified (also called “high yield”) pulp fibers.

**Figure 3:**
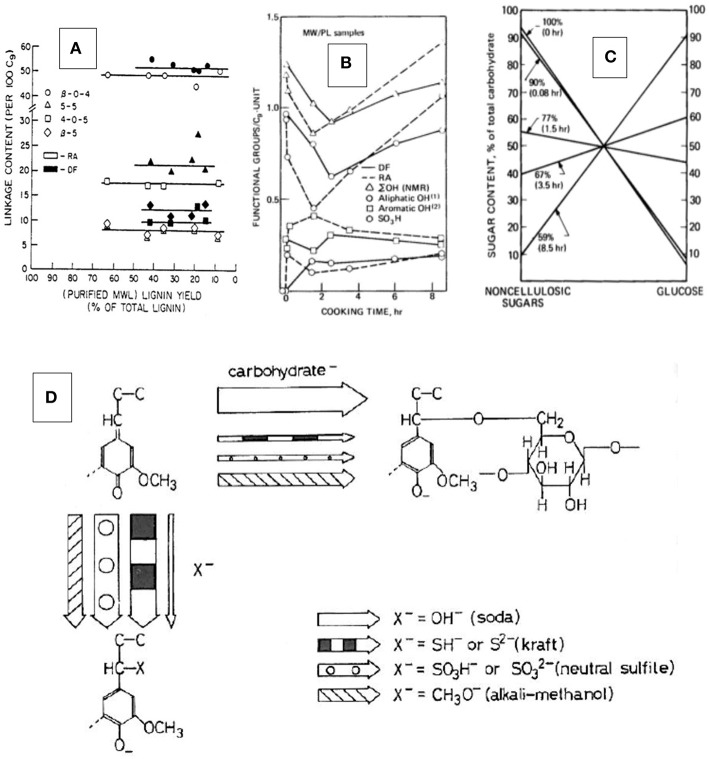
Residual lignin structures in pulp fibers—results of the re-attachment of lignin to carbohydrates in NSSC pulps from Red Alder and Douglas Fir wood (on the basis of ¼ to 8 ½ h of cooking times and representing pulp yields of between 59 and 90%; the recovered, purified and analyzed pulp fiber lignins represented between 6 and 65% of the total lignin content of wood; for more information, see Glasser and Barnett, 1979a): **(A)** Interunit-linkage distribution; **(B)** Functional group distribution (total, aliphatic, phenolic, and sulfonate groups); **(C)** nature of association with carbohydrates (cellulosic vs. non-cellulosic sugar content in % of total carbohydrate content; pulp yield and cooking times indicated on each line; for more information, see Glasser and Barnett, 1979a,b; Glasser and Morohoshi, 1979; Glasser et al., 1979, 1982). **(D)** Proposed mechanism (Glasser, 1981) of the reaction leading to a restored lignin-carbohydrate bond as revealed by Ohara et al. (1980) on the basis of model compound studies.

In a study examining the structure of residual pulp lignins isolated from NSSC-pulp fibers that had been exposed to neutral sulfite pulping conditions for between 5 min (following a 1.33 h presteaming and impregnation time) and 8½ h, the residual lignin content ranged from 6 to 65% of total lignin (Glasser and Barnett, 1979a). The isolated lignin content was thereby inversely related to delignification time. While there were obvious signs of hydrolytic cleavage of weak (lignin-carbohydrate) ether bonds in the first 5 min of cooking (following pre-steaming) with formation of phenolic OH and sulfonate groups on lignin (Figure 3A), the most noticeable change involved the loss of aliphatic OH- groups (Figure 3B) and the formation of new lignin-carbohydrate bonds (Figure 3C) (Glasser and Barnett, 1979b). The residual lignin isolated from the pulp fibers by the Bjoerkman protocol (milled wood lignin) (Bjoerkman, 1956) revealed progressive attachment to cellulosic, and detachment from non-cellulosic carbohydrates with delignification time (Figure 3C). This reversed the initial linking of lignin to, almost exclusively, the non-cellulosic hetero-polysaccharides (“hemicelluloses”). A comparison of the structure of untreated wood lignin with that isolated from the pulp fibers suggested virtually no change in intermonomer bonding (Glasser and Morohoshi, 1979), except for the initial (most likely benzylic) ether hydrolysis with sulfonation (Figures 3A,B). Resistance to delignification coincided with significant changes in functionality, especially the loss of aliphatic hydroxyl groups (Glasser et al., 1979), and to the re-established bonding with (progressively cellulosic) carbohydrates (Glasser and Barnett, 1979b). This made delignification appear like a peeling-off phenomenon without obvious lignin degradation in terms of inter-monomer bonds prior to lignin dissolution. The formation of new lignin-carbohydrate bonds has been attributed to quinonemethide structures in lignin (Glasser, 1981), a conclusion consistent with model compound studies by Ohara et al. (1980) (Figure 3D) and Leary et al. (1983). The block-copolymer nature of the amorphous component of wood was thereby preserved, but the nature of the association of residual lignins with polysaccharides reflected progressively the more abundant (crystalline) cellulose. This progressive detachment/re-attachment created the misleading impression of lignin structure in fiber solids as being variable, with some fractions of lignin resisting dissolution while others dissolve rapidly.

In separate studies on the behavior of lignin during kraft pulping, similar observations were made (Glasser et al., 1983b). Isolated lignin revealed signs of hydrolytic depolymerization to lower molecular weight structures with lower alkyl-aryl ether and higher phenolic OH-contents, whereas residual lignin continued to resemble the native, untreated form. When (commercial) isolated (pine) kraft lignin was fractionated according to molecular size, it became apparent that degradation strictly followed alkyl-aryl ether cleavage to different extents; high molecular weight lignin fractions were found with partially preserved ether bonding, whereas low molecular weight fractions were enriched in C-C bonding and phenolic OH-content (Glasser et al., 1983b).

The analytical procedure used in these studies, known as permanganate oxidation, follows a protocol adopted from earlier work by Freudenberg (Freudenberg et al., 1936), and later by Larsson and Miksche (1967), and that relies on the treatment of lignin (isolated from, or embedded in pulp fibers) with diethylsulfate, an effective ethylating agent for phenolic hydroxyl groups; subsequent oxidative hydrolysis with alkaline cupric oxide; followed by methylation with dimethylsufate; and oxidation of non-aromatic lignin entities with permanganate and hydrogen peroxide (Morohoshi and Glasser, 1979). The resulting mixture of aromatic carboxylic acids is quantitatively separated by gas and gel chromatography following esterification with diazomethane. The ratio of ethyl- to methyl-ethers among the carboxylic acids produces a “hydrolysis factor” that expresses (quantitatively) the degree of alky-aryl ether bonding of the identified aromatic carboxylic acids. Experimental hydrolysis factors were found to range from 0.1 to 1.0 representing alky-aryl ether contents in the range of 10 to 65 per 100 C_9_-units (Glasser et al., 1983a,b).

These results and observations suggest that the variability of native lignin in wood is limited to differences between middle lamella and cell wall lignin; to differences between juvenile and mature plant tissue; and to differences in function (bonding as opposed to stiffening needs) (Monties, 2003). The lack of variability in wood lignin had been a major point of contention in the lignin field in the 1960's and 1970's based on findings by Forss et al. (1966) that seemed to indicate the existence of a “repeating unit structure” in lignin. While this hypothesis (Forss, 1968) failed to find general acceptance (Forss and Fremer, 2000) based on advancing analytical techniques, the conclusion that native lignin in wood has limited variability, and that significant variability is created only by the isolation procedure, gained significant evidential support (Forss and Fremer, 2003).

Much of research (and development) with lignin continues to be carried out with non-industrial preparations—such as experimental fractions derived from mild wood treatment (incl. organosolv and all non-chemical isolations, like milled wood lignin, MWL); from selected lignin fractions (i.e., solvent-soluble fractions of isolated kraft lignins); and from experimental laboratory and pilot plant isolations–which focuses on a wide variety of different lignin structures (Glasser et al., 1983b). This variability, however, is misleading, since isolation on industrial scale is likely to be carried out under highly consistent and invariable conditions, which generate isolated lignins that may vary from pulp mill to pulp mill, and from separation protocol to separation protocol, but that remain invariable when isolated using an invariable delignification procedure.

Since the industrial separation of lignins from wood had the generation of pulp fibers as sole objective for many generations, the isolation process was designed to assist to the utmost degree in the preservation of polysaccharide yield, structure, purity, and size (Rydholm, 1965; The Ljungberg Textbook, 2008). This focus helped generate paper fibers in high yield, with high brightness, in high strength, and with high molecular weight. The goal was achieved by the use of acids or bases in aqueous mixtures. An inexpensive and effective acid was based on sulfur dioxide forming bisulfite and/or sulfite ions in combination with inexpensive bases, such as Ca, Mg, NH4, or Na. This mixture converts lignin into a water-soluble derivative, “lignin sulfonate” (also called “lignosulfonate”), in which many of the native structural details of lignin, mostly the alkyl-aryl ether bonds, remain largely intact. This has led to the utilization of sulfonated lignins from sulfite pulping as water-soluble surfactants and dispersants in applications exceeding one million tons per year worldwide (Gargulak and Lebo, 2000). Pulp production using the “sulfite process” has, however, declined globally due mostly to environmental concerns. It has been replaced in most parts of the world with alkaline processes. While delignification with aqueous sodium hydroxide solutions is effective with herbaceous plants, woody plants resist lignin release (into water) in the absence of a suitable catalyst capable of hydrolyzing alky-aryl ether inter-monomer bonds. The almost universally (but not exclusively) used catalyst in alkaline pulping is the sodium salt of hydrogen sulfide, Na_2_S, which promotes the cleavage of alkyl-aryl ethers in lignin with the simultaneous generation of new solubilizing acidic (phenolic) functionality (Figure 2). Since the alkali-soluble form of lignin can easily be concentrated in water to a level allowing incineration with heat and chemical (ash) recovery, the NaOH/Na_2_S based pulping process (called “Kraft” process) has become worldwide norm. Alternatively, alkali-soluble lignin can be recovered in solid form by the adjustment of solution-pH and filtration. In contrast to lignin sulfonates, which become water-soluble as sulfonated lignin derivatives at any pH, alkali (Kraft) lignin becomes soluble in alkali only, and is insoluble under neutral aqueous conditions and in most organic solvents, due to the creation of acidic (phenolic) functionality by alkyl-aryl ether hydrolysis, and depolymerization (Figure 2). Reduction of pH thus allows Kraft lignin recovery in powder form. Although this lignin has been known, and has been commercially isolated for decades in at least one commercial installation, it has been attracting attention as an optional sustainable, and biodegradable polymer resource only in the last couple of decades.

As stated above, isolated Kraft lignin is inhomogeneous in terms of molecular size and structural functionality depending on its isolation protocol. While some of its alkyl-aryl ether linkages are preserved; new carbon-to-carbon bonds are created; and thiol (mercaptan) functionality is introduced. With the loss of ether bonds, thermal properties are affected with a significant rise of the glass-to-rubber transition temperature (T_g_), and a loss of solubility in all but alkali.

This change, however, is not irreversible. It has been known for several decades that some of the thermal and solution properties of Kraft lignin can be recovered, and lignin can be converted into materials that take advantage of its native design features, bonding and stiffening (modulus-building) (Falkehag, 1975; Glasser and Sarkanen, 1989; Lora and Glasser, 2002). This is the subject of the remaining review.

**Figure 4:**
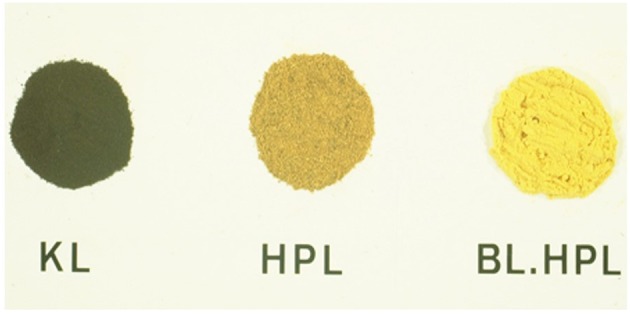
Lignin and lignin derivative color—Typical color of phenolic Kraft lignin (KL) and non-phenolic hydroxypropyl kraft lignin, non-bleached (HPL), and peroxide-bleached (BL. HPL). (According to Barnett and Glasser, 1989).

Lignin in wood is usually a pale-yellow substance. Its isolation under alkaline conditions creates a severe discoloration due to the formation of quinoid structures by phenol oxidation. This discoloration is reversible following modifications that remove phenolic and quinoid functionality (Figure 4) (Barnett and Glasser, 1989).

## Lignin Applications in Materials

The bonding and stiffening attributes of the different forms of isolated lignins have been harnessed in man-made polymeric materials for many decades with varying success. Owing to lignin's character as small, mostly spherically-shaped, multifunctional molecule, the longest and most intensively investigated application has been the use in thermosetting materials in general, and specifically in phenolic resins for wood bonding (Hemingway and Conner, 1989).

While structurally and performance-wise comparable to phenolic (phenol-formaldehyde, PF) network polymers, as additive to phenolic resins lignin has faced the obstacle of variability, dark color, slow cure rate, and lack of chemical reactivity. Isolated (commercial) lignins often resist thermal deformation attempts unless they involve temperatures close to the onset of lignin's thermal degradation (about 200°C). The resulting resins are frequently limited to low rates of addition and extended cure times (Lewis and Lantzy, 1989). The handicap of the limiting solubility and thermal deformability of isolated commercial (Kraft) lignins has been demonstrated to be reversible to different extents by chemical modification. Lignins from non-commercial, experimental sources, however, such as organosolv or steam explosion lignins, with an abundance of T_g_-lowering intermonomer ether bonds, can offer lower T_g_'s as well as more compatible chemistry (Glasser, 2000).

**Figure 5:**
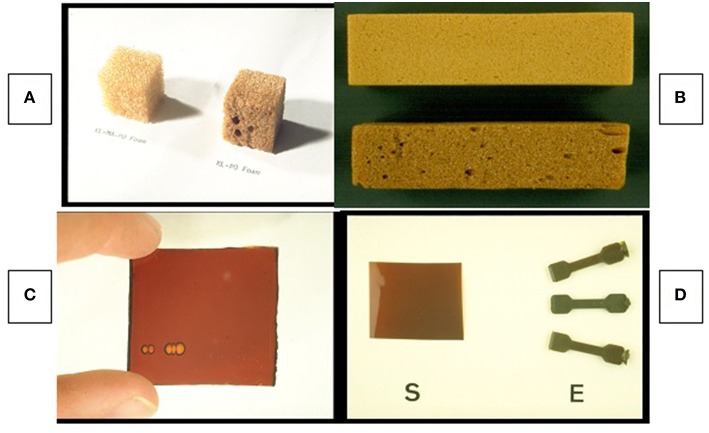
Early lignin-containing structural materials—Polyurethane foams using bleached and unbleached HPL (see Figure 4) according to Hsu and Glasser (1975) **(A)**, and foams according to Lora and Glasser (2002) **(B)**; polyacrylate sheet **(C)** according to Wang and Glasser (1989); and solvent-cast polyurethane coating (S) and injection-molded test specimens (E) according to Ciemniecki and Glasser (1989) **(D)**.

Other applications in thermosetting polymeric materials have included a range of network-forming polymers crosslinked using isocyanate, polyamine, polyacrylate, epoxy etc. resin chemistry (Glasser, 1989; Wang and Glasser, 1989). Several examples of such applications are illustrated in Figure 5. In applications that rely on resin formulations using non-alkaline or non-aqueous conditions, chemical modification or molecular fractionation becomes mandatory since most industrial lignin sources are insoluble in most common solvents. The same is true for applications in thermoplastic materials, where thermal processability requires the restoration of moderate T_g_'s.

**Figure 6:**
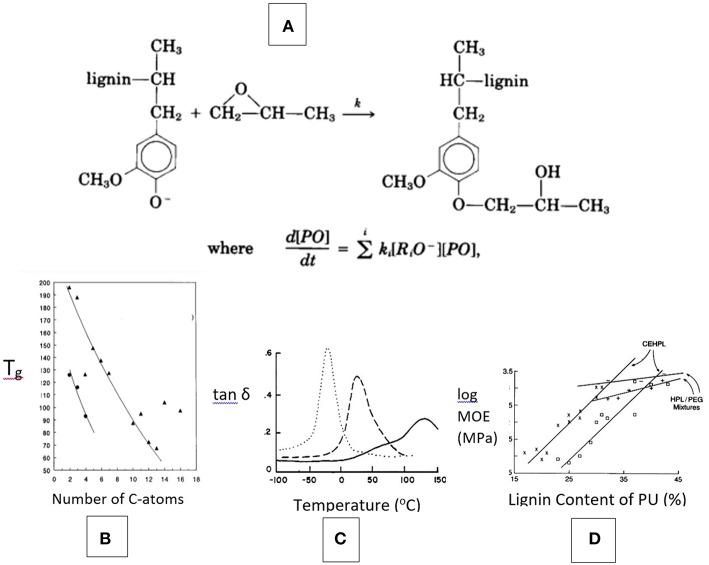
Benefits of lignin modification by propoxylation—**(A)** Derivatization reaction is oxyanion-dependent; **(B)** T_g_ of lignin esters is related to the size of the acyl substituent (i.e., number of C-atoms), applied to Kraft and organosolv lignin with data from Lewis and Brauns (1947), Glasser and Jain (1993); **(C)** Damping (tan δ) thermograms (by DMTA) of polyurethane films containing lignins with variable degree of propylene oxide-chain extension (Kelley et al., 1988); and **(D)** Modulus of polyurethane films with chain-extended hydroxypropyl lignin (CEHPL) compared with HPL/polyethylene glycol (PEG) mixed solutions in relation to lignin content.

The potential recovery of lignin as a solvent-soluble polymer with improved solubility and reduced T_g_ by chemical modification must be considered the first challenge to the reclamation of its performance attributes (Wang et al., 2016; Mueller et al., 2019). It has long been established that the branched nature of lignin combined with its abundance of phenolic OH groups (following isolation involving alkyl-aryl ether cleavage) that give rise to strong internal hydrogen-bonds, is most effectively changed by oxyalkylation as shown in Figure 6A (Hsu and Glasser, 1975, 1976; Glasser et al., 1982; Glasser and Leitheiser, 1984). Non-phenolic hydroxyalkyl lignin derivatives are recognized as virtually uniformly functional (with aliphatic OH-groups) branched molecules with good solubility and thermal properties. Their aromatic character offers many opportunities to contribute to the properties of multi-phase materials that resemble those of natural lignocellulosic materials.

Following chemical modification with alkylene oxides (Figure 6), lignins become sufficiently soluble to perform well in wood adhesives when crosslinked with isocyanates (Newman and Glasser, 1985) as well as in fire resistant foams (Glasser and Leitheiser, 1984). The former has been accomplished, for example, with rapidly curing wood adhesives using blocked diisocyanates as crosslinking agents (Gillespie, 1985). Blends of hydroxypropyl lignin with soy protein have resulted in materials with significantly increased tensile strength and undiminished elongation at break due the formation of supramolecular domains and strong inter-molecular adhesion (Wei et al., 2006).

Combining (non-phenolic) lignin derivatives with such inherently low-modulus polymer components as polyether glycols mimics the role lignin plays in the amorphous component of wood, where it adds modulus by the formation of copolymer structures with gum-like non-crystalline hetero-polysaccharides (Kelley et al., 1988, 1989). This has been examined repeatedly for the case of both lignin-containing mixtures with, and copolymers of, polyaliphatic glycols (Figure 6). Whereas addition of an extended aliphatic polyether glycol chain to lignin (CE-HPL in Figure 6D) by chemical modification provides flexibility in terms of thermal and mechanical properties of a polyurethane-based network polymer, simple mixing of Kraft lignin derivatives with aliphatic glycols (HPL/PEG in Figure 6D) prior to crosslinking helps introduce a more moderate, but still noticeable, and effective modulus-raising aromaticity to the cured resin structure as well (Moerck et al., 1989). Such mixtures of aromatic and aliphatic polyols have been demonstrated to become the basis of a variety of adhesives, coatings, foams, etc., in which the inherent properties of lignin are adopted for achieving mechanical, thermal, moisture absorbing, sustainability, and other property goals (Figure 5).

These examples demonstrate that lignin as isolated by separation from woody biomass performs like a normal polymer in a predictable manner and continues to present the option of re-use in a form it was initially designed for by nature.

In addition to thermosetting materials, lignin can also contribute important properties to thermoplastic mixtures (polyblends) with other polymers as long as it meets thermal and compatibility needs. Lignin in native state, such as in solid wood, will readily undergo thermal deformation if (a) the plasticizing effect of water on amorphous hetero-polysaccharides is assured, and (b) the T_g_ of (water-plasticized) lignin is exceeded (Ito et al., 1998). This has been demonstrated at temperatures of 135–150°C since lignin's *in-situ* T_g_ has been reported to be around 60–80°C (Figure 1). The Tg of lignin rises with loss of alkyl-aryl ether bonds to >180°C (Falkehag, 1975); it can be restored by suitable chemical modification, most commonly by esterification or etherification (Figure 6).

Thermoplastic blend materials with lignin (derivatives) having acceptable T_g_'s have been widely demonstrated. Ester formation has been studied in terms of the effect on thermal properties as early as 1947 (Lewis and Brauns, 1947; Glasser and Jain, 1993), and etherifications by ring opening reactions with aliphatic oxirans have been pursued by Glasser et al. (Wu and Glasser, 1984; Jain and Glasser, 1993).

For reasons of biodegradability and sustainability, thermally processable natural polymers have attracted attention also in the field of melt-processed materials. Since ester-type lignin derivatives meet those requirements, their feasible incorporation into blend materials has been examined (Ghosh et al., 1999, 2000). Lignin esters were found to be readily miscible with cellulose esters, starch esters and poly(hydroxybutyrate).

Because of its potential contribution to the modulus of rigid multi-phase materials and its resistance to thermal degradation, lignin has also been considered as an attractive source for carbon fibers (Fukuoka, 1969). However, because of its inability to organize in melt-state into liquid crystalline condition, an attribute of benefit for lignin's function in lignocellulosic composite structures, lignin appears to have a limiting effect on building strength in carbon fibers (Davé et al., 1993).

## Isolated Lignin's Limitations

**Figure 7:**
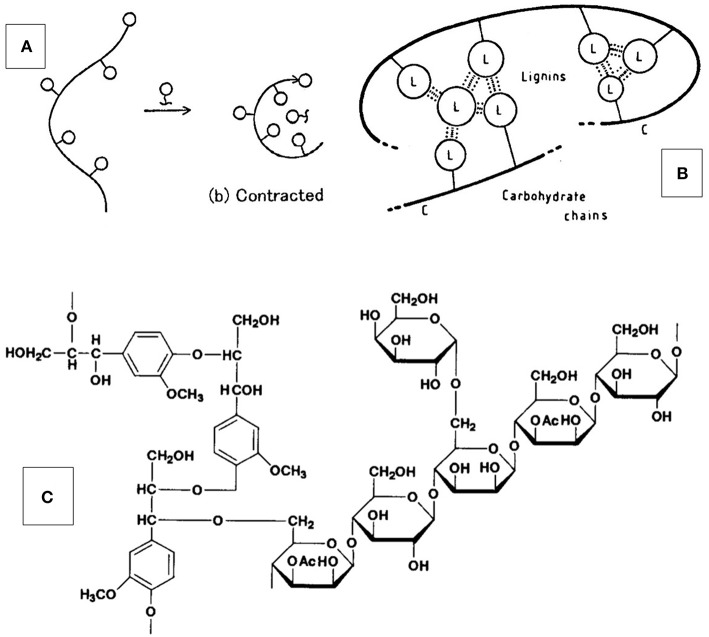
Model of molecular structure and architecture of the amorphous component of wood— **(A,B)** Concept of lignin-carbohydrate copolymer (LCC) self-associating (“contracting”) with lignin-rich phases via secondary bonds (on the nano-scale); this effect of copolymer structure on solution behavior has been studied using LCC-model copolymers by Esker et al. (see Gradwell et al., 2004; Westbye et al., 2007; Kaya et al., 2009); **(C)** Schematic lignin-carbohydrate complex with benzyl ether linkage between lignin and branched xylan.

Lignin appears to be a relatively small molecule, both *in vivo* and *in vitro*, that serves lignocellulosic multi-phase materials (i.e., cellular tissues of plants) exclusively as a component of a block copolymer with amorphous hetero-polysaccharides (also called “hemicelluloses”). Figures 1C and 7 represent widely–used illustrations of the molecular architecture of the amorphous component of wood. Since thermal and solubility characteristics of the two components, hetero-polysaccharides and lignin, are very different, the copolymer behaves like a crosslinked, three-dimensional network structure in native (unplasticized) wood. The polysaccharides provide stability and immobility in dry as well as high-temperature state, and lignin anchors the branched (gum-like) hetero-polysaccharides in place under high-moisture conditions. It can therefore not be expected that lignin by itself will qualify as stand-alone polymeric material. It is most likely performing best as partner with a molecular entity that complements its properties, as it does in wood. This partnership must, by design, experience different levels of stress that results in the separation of molecular components into individual phases. Such molecular stresses are relieved when wood is exposed to high temperature moisture, such as during “steam explosion” treatments involving exposure to high-pressure steam for short time periods (Focher et al., 1991). The molecular response involves (a) lignin-carbohydrate separation by hydrolysis, and (b) relocation of the separated lignin within the fibrous tissue (Debzi et al., 1991). Depending on the severity of the steam treatment, other hydrolytic and/or condensation reactions are encountered that change lignin's functionality and molecular character. However, it is obvious that disruption of the block copolymer architecture of the amorphous component of wood results in spontaneous phase-separation of carbohydrates and lignin within the limits of mobility. This results in the opportunity to extract lignin partially with aqueous alkali or organic solvents without further pretreatment (Wright and Glasser, 1998). The re-incorporation of lignin into polymeric materials structures (i.e., the “making lignin great again”-effort), in networks or blends, thermosets or thermoplastics, requires “phase compatibilization.” In either case, thermosets or thermoplastics, the molecular mixture proceeds toward solidification via a homogeneous liquid phase at either ambient or elevated temperature. Phases separate on either or both, kinetic and thermodynamic grounds.

**Figure 8:**
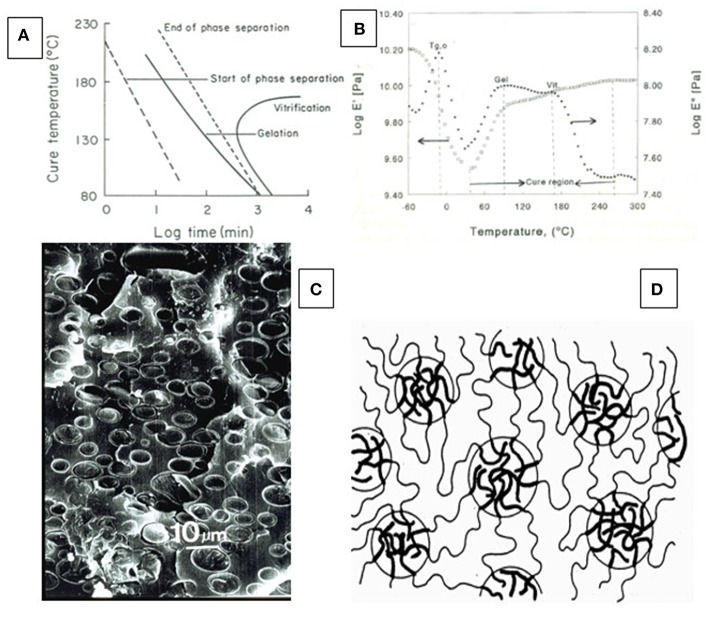
Basis of phase separation in lignin-containing thermosets—**(A)** Schematic time-temperature-transformation (TTT)-diagram identifying phase separation preceding gelation and vitrification on a time scale (after Gillham, 1986); **(B)** Dynamic mechanical thermal analysis (DMTA) diagram of a lignin-containing thermosetting resin mixture undergoing cure with identification of gelation (gel), and vitrification (vit) on the temperature-scale (Toffey and Glasser, 1997); **(C)** SEM picture of cured lignin-containing thermoset with phase separation of (presumably lignin-rich) particulate inclusions (Hsu and Glasser, 1976); **(D)** Schematic phase separation of gel-forming molecules illustrating the clustering that produces regions of greater mass density embedded in a continuous phase of lower-density gel (after Kinloch, 1986).

Lignin's potential as component in structural materials, both network-forming and thermally processable, is limited by its solution and thermal properties. Restricting the solubility of isolated Kraft lignin to aqueous alkali, and its thermal deformability (i.e., glass transition temperature, T_g_) to close to its thermal degradation temperature, leaves little opportunity for incorporation into multi-phase polymeric network materials systems. The degree to which such limitations apply to thermosetting materials has been analyzed by Gillham using a time-temperature-transition (TTT)-diagram (Figure 8) (Gillham, 1986); and thermoplastic blend compatibility is often analyzed in terms of thermodynamic solubility characteristics (Rials and Glasser, 1989a,b, 1990). Both approaches have been applied to lignin-containing polymeric materials.

The TTT-diagram records molecular changes of resin mixtures under cure by isothermal heat exposure on a time scale. During isothermal heating of homogeneous thermosetting mixtures at different temperature-levels, three events take place in sequence: (a) separation of molecular phases representing differences in chemical or molecular structures (incl. differences in molecular origin or differences in crosslink density, Figure 8C) (i.e., “demixing”); (b) formation of a continuous phase by gelation; and (c) gel densification (“vitrification”) with the formation of additional crosslinks resulting in a rise in T_g_ to the point where T_g_ reaches cure temperature (T_c_) (Figure 8). This process can, in part, be followed experimentally by thermal analysis (differential scanning calorimetry, DSC, and/or differential mechanical thermal analysis, DMTA) for many network polymers (Figure 8B) (Toffey and Glasser, 1997).

Using these analytical principles, it becomes apparent that an excessive separation of phases prior to gelation can produce particulate-filled polymer networks in which the included particles are rubbery or glassy; and the sizes are on the nano-, micron-, or millimeter-scale. In resin mixtures that contain molecules with different character (i.e., functionality, solubility parameter, size, etc.), phase compatibility undergoes a dynamic change as gelation progresses with the consequence of separation. This is illustrated in Figure 8D (Kinloch, 1986). In the case of a resin mixture with lignin, phase separation likely results in the formation of glassy (because of high T_g_) particles disconnected from the (continuous) network polymer. This formation of a particulate “composite” architecture representing solids dispersed in a continuous polymer matrix must not be detrimental to performance unless the enclosed particles exceed a certain concentration or size. It must be recognized that this formation of an inhomogeneous resin phase depends on both lignin functionality and molecular size, with lower molecular fractions preserving phase compatibility for a greater period during the cure cycle. The employment of low molecular weight lignin fractions is therefore preferable in phenol-formaldehyde (PF) resin products (Gillespie, 1985).

An example of a phase-separated polyurethane network with lignin-rich particulate enclosures is given in Figure 8C. Even though the component mixture, polyol and isocyanate, started the curing process as homogeneous mixture, it quickly experienced phase separation as gelation advanced. This phenomenon is observed universally with network forming mixtures, regardless of chemistry. In the case of lignin, it can be retarded by chemical modification aimed at the development of structural compatibility, or by the addition of functionality (chemical modification), that promotes the participation in crosslinking (gelation) reactions before phases separate. Different resins have different tolerances (in terms of molecular size and chemical functionality as well as cure cycle times) to the formation of separate phases during normal gelation.

**Figure 9:**
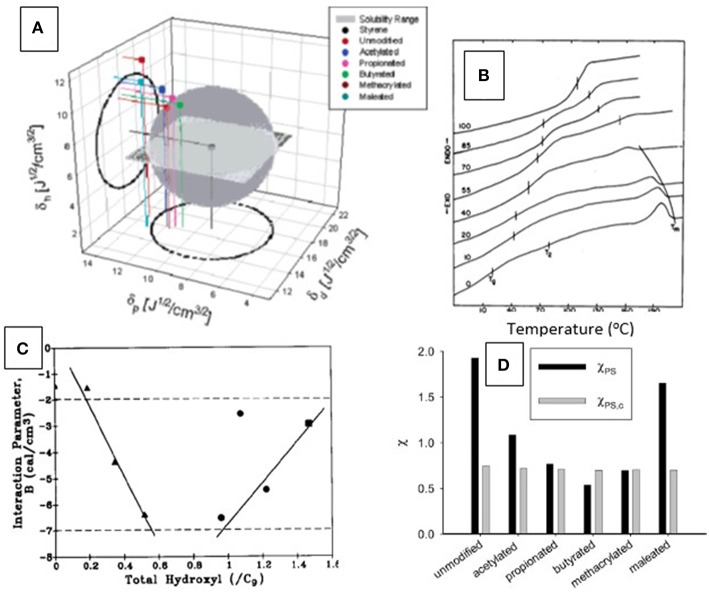
Analysis of phase compatibility—**(A)** Theoretical range of solubility parameters of several (Kraft) lignin esters as predicted by the Hoy model, compared to the solubility range of styrene (from Thielemans and Wool, 2005). Only the points representing the lignin derivatives fitting into the gray sphere can be considered phase compatible with polystyrene. **(B)** Differential scanning thermograms (DSC) of hydroxypropyl cellulose blended with 0 to 100% hydroxypropyl lignin (bottom to top) revealing single T_g_'s and indicating phase compatibility (adopted from Rials and Glasser, 1990). **(C)** Relationship between experimentally determined Flory-Huggins polymer-polymer interaction parameter, B, of two (organosolv) lignin derivatives [acetate (◦), and ethyl ether, (Δ)] and lignin total hydroxyl group content in solvent-cast blends of lignin derivatives and hydroxypropyl cellulose illustrating the importance of optimal lignin functionality on phase compatibility in blends. Phase compatibility rises with the negative magnitude of the B-parameter. Maximum compatibility is reached with lignin derivatives having OH-contents of 0.8 per C_9_-repeat unit, similar to that of native lignin (from Rials and Glasser, 1990). **(D)** Comparison of the calculated Flory-Huggins interaction parameters χ_PS_ of lignin esters with the critical value, χ_PS,c_, derived from the ratios of molar volumes of polymer and solvent (polystyrene). Compatibility is reached if χ_PS_ < χ_PS,c_. In this graph, only lignin butyrate is predicted to be miscible with polystyrene (from Thielemans and Wool, 2005).

Similar arguments can be applied to the analysis of phase separation in thermoplastic polymer mixtures (i.e., polyblends), illustrated in Figure 9. This has previously been approached experimentally with lignins modified in different ways and to different extents prior to solution-blending with non-crystalline (thermoplastic) cellulose derivatives (Figures 9B,C) (Rials and Glasser, 1988, 1990). The results of this study demonstrated that a difference in solubility parameter between lignin and cellulose derivatives was responsible for the formation of regions with greater or lesser molecular compatibility. Calculations based on experimental observations of the shift in T_g_ with blend composition (Figure 9C) resulted in the conclusion that greatest phase compatibility between the derivatives of cellulose and lignin (i.e., most negative polymer-polymer interaction parameter, B) was achieved when the lignin derivative had a free OH-group content comparable to that in wood (i.e., 0.8/C_9_) (Rials and Glasser, 1989a) (Figure 9C).

Phase compatibility of lignin derivatives with polystyrene has also been assessed using a theoretical approach involving solubility parameter calculations (Figure 9A). Various chemical modification treatments of lignin were found to allow predicting the compatibilization of lignin derivatives with polystyrene (Thielemans and Wool, 2005). Esters of lignin with selected acids were found to contribute to both compatibility and reduced thermal transitions (Figure 9D).

## Compatibilization by Functionalization

Creating single-phase network structures with lignin requires both component miscibility and complementary functionality. This approach is routinely pursued by the attachment of (a) reactive functional groups, such as phenols (by “phenolation”), acrylates, epoxies, amines, vinyl, etc; or (b) by modification with molecular entities that promote the formation of secondary interactions, such as hydrogen bonds, with the surrounding (continuous) polymer phase (Thielemans et al., 2002; Bova et al., 2016; Kun and Pukanszky, 2017).

The desire to use lignin in phenol-formaldehyde (PF) resins has an extremely long history (Lambuth, 1989). It resurfaces any time the price of phenol rises (Lake, 2016). Both, lignin sulfonates and kraft lignins, are amenable to substituting phenol; but this requires either molecular fractionation or chemical modification of the parent lignin if high substitution degrees are to be reached. The most common derivatization involves methylolation by reaction with formaldehyde and/or phenolation (Muller and Glasser, 1984; Hemingway and Conner, 1989).

**Figure 10:**
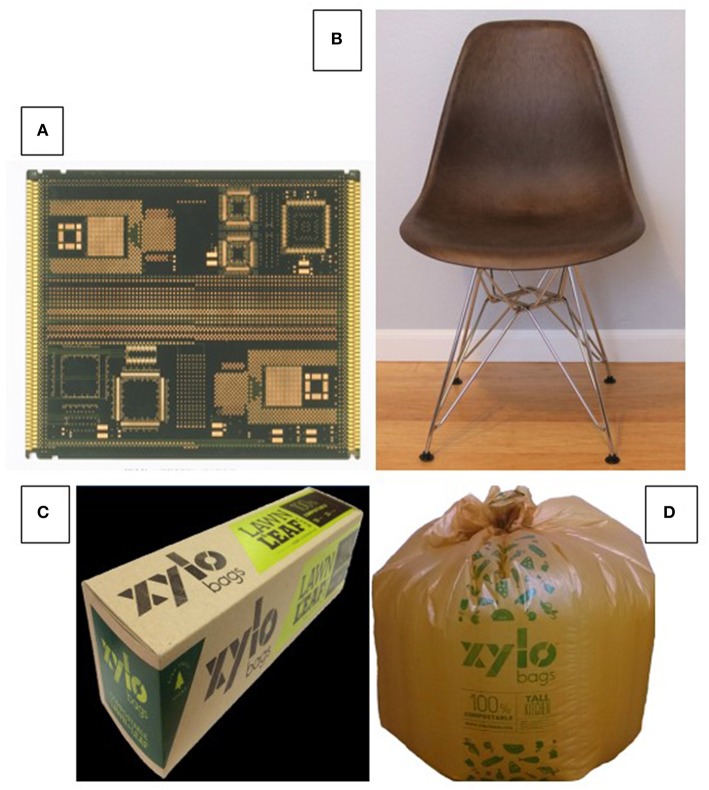
(Semi-) commercial products with lignin—**(A)** Prototype printed circuit board manufactured with glass fiber-reinforced (organosolv) lignin-containing (50%) epoxy resin (IBM pilot plant) (Kosbar and Gelorme, 1996; Shaw et al., 1996; Kosbar et al., 2001); **(B)** Prototype shell of injection-molded chair made from polypropylene/lignin derivative blend; and **(C,D)** commercial compostable waste bag made with melt-blown films of blends of hydroxypropyl (Kraft) lignin derivative and biodegradable polyester (*Ecoflex* by BASF) (Glasser et al., 2017).

Another example of phase compatibility gained by functionalization is shown in Figure 10A, which represents a prototype printed circuit board based on glass fiber-reinforced epoxy resin containing 50% lignin (Kosbar and Gelorme, 1996; Shaw et al., 1996; Kosbar et al., 2001). Lignin content in epoxy resins can be achieved using several approaches. The functionalization of lignin with oxiran functionality produces resins crosslinkable with amines or phenols (Nieh and Glasser, 1989; Hofmann and Glasser, 1993; Lora and Glasser, 2002), whereas bis-phenol A-based epoxies are capable of serving as crosslinking agents for phenolated or aminated lignin (derivatives).

The introduction of functionality useful for participation in chemical reactions is limited only by sufficient molecular compatibility to prevent premature phase separation, as pointed out above. Like other (natural) polymers, like cellulose and starch for example, the solubilizing and/or plasticizing effect of substituents can be generalized (within limits) based on character and degree of substitution.

In polyblend applications, in which a lignin component is mixed in homogeneous melt-state with a thermoplastic polymer, both thermodynamic and kinetic factors dictate the degree of phase separation. Examples of blends of (lightly modified) lignin with thermoplastic polymers in need of stiffening are shown in Figures 10B–D, which represent an injection-molded chair seat with polypropylene (Figure 10B), and a melt-blown waste bag made with a biodegradable polyester (Figures 10C,D) (Glasser et al., 2017). Improving the solubility and thermal properties of isolated (Kraft) lignin thereby helps to compatibilize the two components sufficiently (but incompletely) to assure mutual support. This is a prerequisite for opening important pathways to lignin's use in melt-blended polymeric materials based exclusively on secondary molecular interactions.

Using nature's approach to the design of greater component compatibility required from inherently (thermodynamically) incompatible polymer types employs the creation of co-polymers that help in the dispersion of otherwise incompatible but (structurally) complementary molecular phases. In the case of natural lignocellulosic materials, kinetic phase compatibilization factors are eliminated, and thermodynamic factors become prevalent.

Mimicking the approach of compatibilizing an aromatic polymer (lignin) with a crystalline polysaccharide (i.e., cellulose) on the nano-scale, without ever forming covalent bonds between the blend components, as shown in Figure 7, has been adopted for lignins compatibilized with thermoplastic cellulose esters, polystyrene, and poly(vinyl chloride) (PVC) (de Oliveira and Glasser, 1989). In all cases, co-polymer synthesis was achieved by reacting mono-functional (with terminal NCO-groups) synthetic polymer segments (of variable sizes) with lignin derivatives to produce star-like block-copolymer structures resembling native lignin-carbohydrate complexes. These block-copolymer preparations were solution-blended with the linear polymer partner (de Oliveira and Glasser, 1993, 1994a,b).

**Figure 11:**
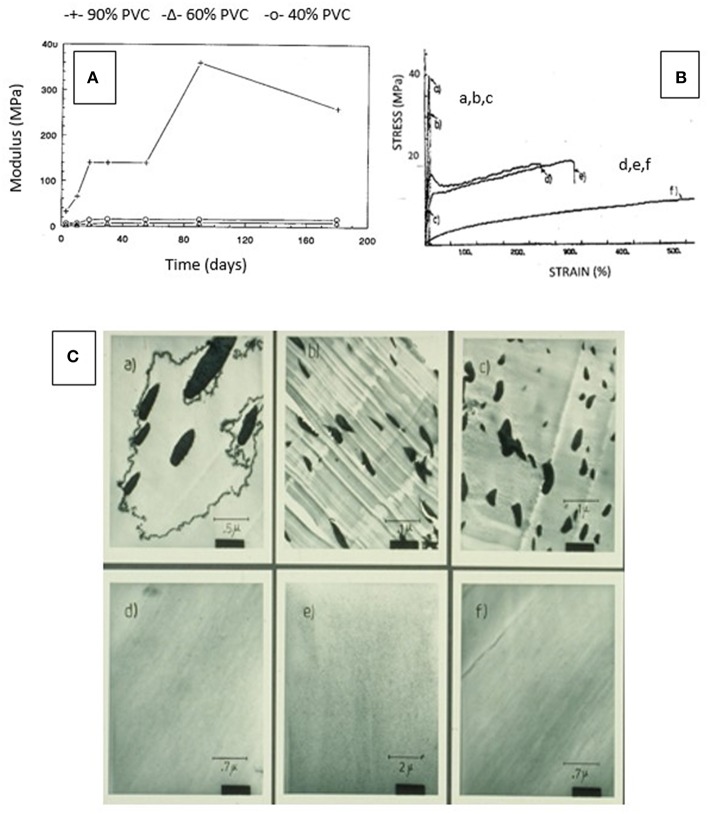
Consequence of phase compatibilization in PVC blends with lignin (derivative)—**(A)** Relationship between modulus and degree of aging (time) for three different lignin derivative/PVC blend formulations (with 10, 40, and 60% lignin content) illustrating the long-lasting plasticizing effect of lignin derivative (de Oliveira and Glasser, 1994b). **(B)** Stress—strain curves of PVC/lignin derivative blends (solvent-cast films) shown in part C: blend formulations a-c reveal brittleness whereas blends d-f display ductile behavior based on phase compatibility (on the nano-level) (de Oliveira and Glasser, 1994b). **(C)** Illustrations by SEM of the degree of compatibility achieved by block copolymer formation of HPL and caprolactone segments (a-c are caprolactone-free, and d-f reveal the impact of the presence of caprolactone as copolymer component). (de Oliveira and Glasser, 1994b).

The response of changes in blend properties to composition was remarkable in terms of mechanical, thermal, crystallinity, and morphological characteristics as is illustrated for PVC blends in Figure 11 (de Oliveira and Glasser, 1994b). The compatibilizing power of caprolactone attached to lignin resulted in blend compositions that revealed either miscibility or near-miscibility of the aromatic and linear phases. Whereas, blends of (polycaprolactone-free) lignin derivatives with PVC generated structures with macro-phase separation on the scale of 0.1–1.0 μm, the presence of caprolactone revealed copolymer domains sized about 10–30 nm (Figure 11C). Similarly astonishing was the consequence on mechanical properties following aging (Figure 11A), and on stress- and strain-levels (Figure 11B) (de Oliveira and Glasser, 1994b). Blends with copolymer contents above the 30–40%-level were found not to experience the well-known embrittlement with age usually observed with PVC materials and indicated by a time-dependent rise in modulus and tensile strength (Figure 11A); and the stress-strain behavior of the blends revealed extreme brittleness for all caprolactone-free compositions (Figure 11B).

The compatibilization of lignins for use in thermoplastic polymer systems has found a significant following in recent years. The effect of derivatization with fatty acids of different sizes was found to enhance both phase morphology and melt rheology (Koivu et al., 2016); and the molecular nature of the aliphatic ester substituents of Kraft lignin was found to play a significant role on crystalline and supramolecular complexes of aliphatic polyesters in relation to methylene/carboxylate group ratios (Li and Sarkanen, 2002). Thermoplastic blends of acrylonitrile-butadiene rubber with various lignins in different blend ratios were found to produce tough materials with high failure strains (Bova et al., 2016; Nguyen et al., 2018).

The gradual build-up of lignin-containing phases on cellulose surfaces (mimicking a hydrophobization effect of plant fibers required by native plants) using copolymers of lignin with hetero-polysaccharides has been studied in aqueous environments (Gradwell et al., 2004). “Docking” was registered by surface plasmon resonance spectroscopy, and it was revealed that very low lignin concentrations attached to water-soluble (model) carbohydrates, pullulan and xylan, for example, effectively promote adsorption of these substances on mono-molecular (crystalline) cellulose surfaces (Westbye et al., 2007; Kaya et al., 2009). This suggests that lignin-carbohydrate copolymers serve as phase compatibilizers between cellulose and lignin. It is obvious that this parallels the multi-phase molecular architecture of wood, where a lignin-rich copolymer phase of water-soluble or gum-like non-crystalline polysaccharides becomes attached to cellulose crystals via secondary bonds. The principle of phase compatibilization of lignin and PVC via caprolactone parallels that of lignin-co-hetero-polysaccharides and cellulose. The principle produces non-covalently-linked blends of molecular entities with widely different characteristics on the nano-scale. The principle is adoptable for the development of a virtually unlimited range of sustainable and biodegradable materials.

## Conclusions

1. The structure of native (especially softwood) lignins is highly invariable in terms of its inter-monomer bonding and functionality. Differences are detectable between middle lamella and secondary cell wall location, among other factors.
2. The aromatic character of lignin is compatibilized with crystalline cellulose fiber surfaces by the formation of block copolymers with hetero-polysaccharides (“hemicelluloses”).
3. Isolated lignins vary distinctly regarding their alkyl-aryl ether contents, degrees of secondary condensation, molecular weights, solubility, and thermal properties.
4. Lignins retained by chemical pulps (NSSC and Kraft) exposed to between 5 min (following presteaming and heat-up) and 8½ h to pulping conditions, and representing yields between 6 and 65% of total lignin, are virtually identical to the native wood lignins in structure (esp. inter-unit bonding). Resistance to dissolution is a consequence of re-attachment to polysaccharides (incl. cellulose) via ether bond formation involving quinonemethide intermediates.
5. Since structural variability of lignins is primarily the result of the isolation protocol, commercial sources of lignin isolated by any procedure under invariable process conditions can be expected to remain invariable.
6. Isolated lignin represents an important and abundant biopolymer resource the behavior of which is highly predictable and adaptable based on its varied functionality and modifiability.
7. Structure and properties of isolated lignins are subject to wide-ranging changes via chemical modification. Properties can readily be tailored for uses that require special solubility, thermal and compatibility behaviors.
8. Modified lignins have the potential of contributing the characteristics of lignin in wood to man-made thermoplastic and thermosetting materials, such as bonding, stiffness (modulus), fire resistance, biodegradability, and sustainability.
9. Best results are achieved when lignin's structural modification adopts the principles of its nature in wood by partnering with polymers via compatibilization.
10. A variety of lignin derivatives have been converted into such products as adhesives, coatings, foams, printed circuit boards, melt-blown compostable films and other thermosetting and injection-molded thermoplastic materials.
11. In many cases, such undesirable properties as malodor associated with the isolation method, esp. Kraft pulping, need to be overcome for lignin to reach its inherent “greatness.”

## Author Contributions

The author confirms being the sole contributor of this work and approved it for publication.

### Conflict of Interest Statement

The author declares that the research was conducted in the absence of any commercial or financial relationships that could be construed as a potential conflict of interest.
